# Abdominal Aortic Aneurysm with Primary Cold Agglutinin Disease Treated with Endovascular Aortic Repair

**DOI:** 10.3400/avd.cr.24-00107

**Published:** 2025-01-01

**Authors:** Shinichi Tanaka, Takahiro Ohmine, Ryota Imanaka, Takashi Maeda

**Affiliations:** 1 Department of Surgery, Hiroshima Red Cross Hospital and Atomic-bomb Survivors Hospital, Hiroshima, Hiroshima, Japan; 2 Department of Hepatology, Hiroshima Red Cross Hospital and Atomic-bomb Survivors Hospital, Hiroshima, Hiroshima, Japan

**Keywords:** primary cold agglutinin disease, abdominal aortic aneurysm, endovascular aortic repair

## Abstract

Cold agglutinin disease (CAD) is a rare and autoimmune hemolytic disorder caused by the presence of cold-reacting autoantibodies against red blood cells. An abdominal aortic aneurysm (AAA) is a potentially life-threatening condition. This report describes an 83-year-old man with AAA who was diagnosed with primary CAD 9 years before undergoing AAA surgery. The patient underwent successful endovascular aortic repair. Temporary hemolytic anemia and exacerbation of jaundice were observed postoperatively despite strict temperature control. Red blood cell and haptoglobin transfusions may prevent fatal hemolytic anemia, renal disorders, embolism, and systemic complications.

## Introduction

Cold agglutinin disease (CAD) is a rare disorder and the second most common autoimmune hemolytic anemia (AIHA) that is caused by the presence of cold-reacting IgM autoantibodies against red blood cells.^1^^,^^2)^ Autoantibodies react in a temperature-specific manner and may cause red cell agglutination and complement-mediated hemolysis.^1^^,^^2)^ Although newer drugs, such as rituximab, are effective, CAD treatment is based primarily on protection from cold.^1)^ Particular attention should be given to temperature control during surgery.^3)^ Hypothermia can trigger hemolytic anemia, which can lead to systemic microvascular thrombosis and catastrophic consequences. We report a case in which we successfully performed endovascular aortic repair (EVAR) to treat an abdominal aortic aneurysm (AAA) in a patient with CAD.

## Case Report

**Figure 1:**
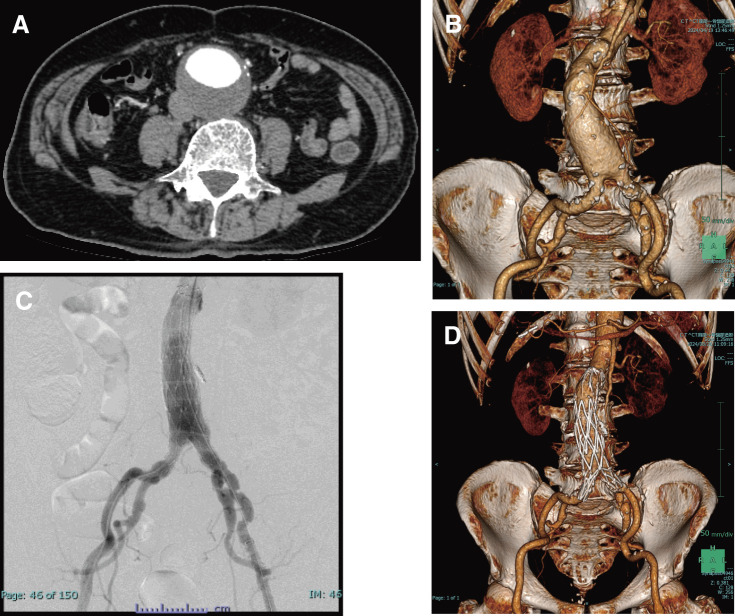
Fig. 1 (**A**) Preoperative enhanced CT showed a 51-mm AAA. (**B**) Preoperative three-dimensional CT. (**C**) The post-EVAR angiogram of the abdominal aortic artery demonstrated preservation of both internal iliac arteries with no endoleak. (**D**) Postoperative CT showed exclusion of the aneurysm preservation of both renal arteries as well as both internal iliac arteries with no endoleak. CT: computed tomography; AAA: abdominal aortic aneurysm; EVAR: endovascular aortic repair

**Figure 2:**
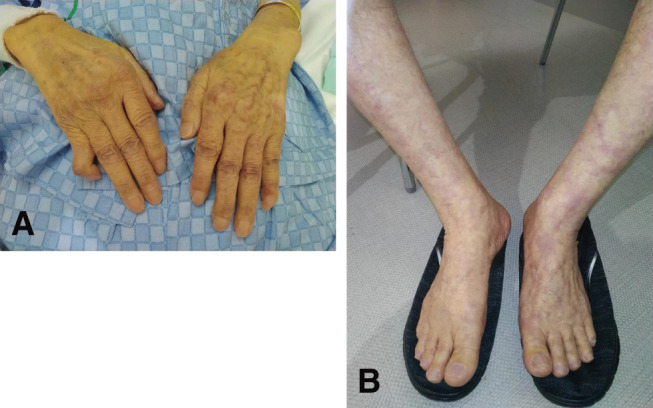
Fig. 2 (**A**) Livedo reticularis of the upper extremities and (**B**) livedo reticularis of the lower extremities during cold exposure.

**Figure 3:**
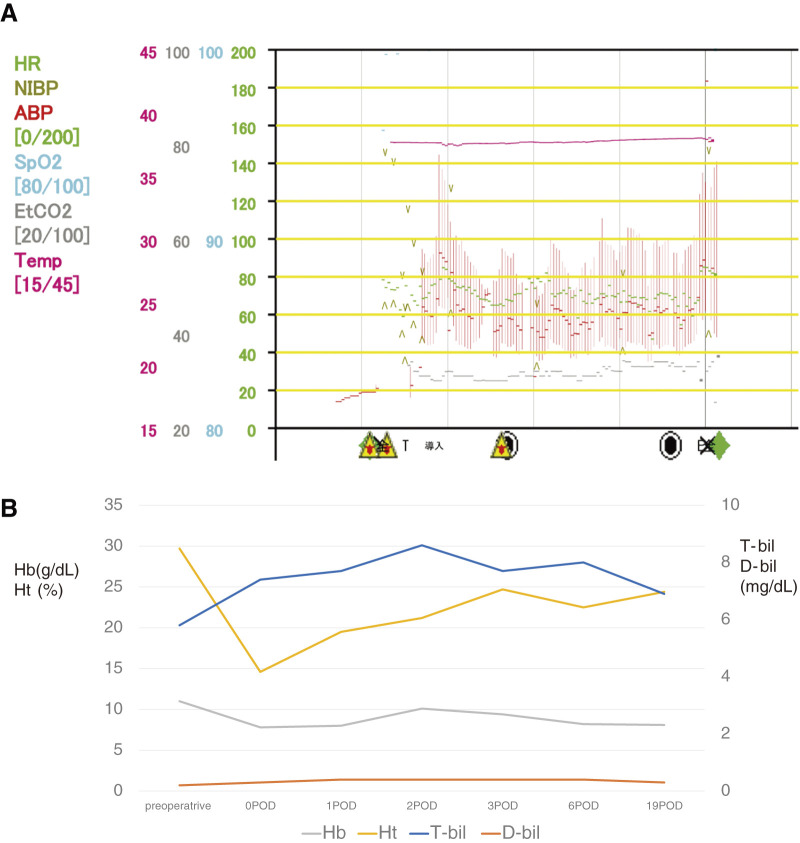
Fig. 3 (**A**) Anesthesia progress records revealed the real-time bladder temperatures during the operation. (**B**) The line graphs show the values of perioperative Hb, Ht, T-bil, and D-bil. T-bil: total bilirubin; D-bil: direct bilirubin

An 83-year-old man was referred to our department for a 51-mm AAA (**Figs. 1A** and **1B**) that was unexpectedly found by computed tomography. Nine years before his referral to our department, he had presented with Raynaud’s symptoms, and he was examined by the hematology department. Blood tests were positive for hemolytic anemia and cold agglutinin, and the patient was diagnosed with CAD; he was followed up in an outpatient clinic. During exposure to cold exposures such as air conditioning, the patient presented with livedo reticularis of the upper and lower extremities (**Figs. 2A** and **2B**). He had jaundice, with total bilirubin values in the 5–6 mg/dL range, due to chronic persistent hemolysis. Surgery was planned in the summer to prevent hypothermia during the perioperative course. The patient underwent EVAR after consideration of his comorbidities, age, and anatomical characteristics of the aneurysm. We warmed the intravenous fluids and also a saline solution for flushing during inferior mesenteric artery embolization before EVAR to prevent a Type 2 endoleak. We performed EVAR using the Endologix AFX2 Bifurcated Endograft System (Endologix, Irvine, CA, USA) under general anesthesia. The temperature of the operating room was adjusted to 29°C. The patient’s upper and lower extremities were carefully covered with pre-warmed blankets. All fluids, including intravenous fluids, irrigation fluids, blood products, and intravenous drugs, were warmed and maintained at 37°C before use. The patient’s core temperature was monitored by the bladder sensors and maintained at 37°C–38°C during his stay in the operating room (**Fig. 3A**). During the surgical procedure, more heparin (80 U/kg) was injected than what is normally used (50 U/kg). The post-EVAR angiogram of the abdominal aortic artery demonstrated preservation of both iliac arteries with no endoleak (**Fig. 1C**). Decreased Hb and Ht levels and increased total bilirubin levels were temporarily observed during the postoperative period (**Fig. 3B**). Red blood cells and haptoglobin transfusion was performed via an in-line blood warmer was on the day of surgery and 1st and 3rd postoperative days. There were no fatal complications, and the patient was discharged on the 7th postoperative day. Follow-up computed tomography confirmed the successful exclusion of the aneurysm with no endoleak (**Fig. 1D**).

## Discussion

Specific precautions are required for CAD if surgical intervention with general anesthesia is needed.^3^^,^^4)^ The primary goal is to avoid hypothermia, resulting in hemolytic anemia and systemic thrombosis during the perioperative course.^3^^,^^4)^ Although CAD has been reported during cardiac and aortic arch surgery,^5^^–^^8)^ to the best of our knowledge, this is the first report of surgery for AAAs with primary CAD in English. First, surgery in winter should be avoided, as with all other surgeries. Second, EVAR should be the first choice for surgical technique selection if the anatomical conditions are favorable, as it allows for less intraoperative blood loss and shorter surgical and anesthesia times than open surgery does.

During the EVAR procedure, more heparin was injected than what is normally used to avoid clot formation. Although there is no clear evidence for increasing the heparin dosage, thromboembolic complications could be avoided in the present case. Despite strict temperature control during the perioperative period, the patient developed hemolytic anemia and exacerbation of jaundice after EVAR. He required transfusions of red blood cells and haptoglobin, after which anemia and jaundice improved. Replenishment of haptoglobin in the blood can reduce renal damage associated with hemolysis by transporting excess free hemoglobin to the liver. Red blood cell and haptoglobin transfusions may prevent fatal hemolytic anemia, renal disorder, embolism, and systemic complications. Patients with CAD require careful perioperative management even during EVAR, which is considered to be lower risk than cardiac or aortic arch surgery supported by systemic hypothermia or cold cardioplegia.^5^^–^^8)^

## Conclusion

We herein report a rare case of an AAA with primary CAD treated with EVAR alongside careful perioperative control.

## Declarations

### Informed consent

The patient provided his written informed consent for the publication of the details of his case.

### Acknowledgments

We thank American Journal Experts (http://bit.ly/AJE-HS) for editing a draft of the manuscript.

### Disclosure statement

All the authors have no conflicts of interest.

### Author contributions

Study conception: ST

Data collection: ST

Analysis: ST

Investigation: ST

Manuscript preparation: ST

Funding acquisition: None

Critical review and revision: All authors

Final approval of the article: All authors

Accountability for all aspects of the work: All authors.
